# M2 macrophage-derived soluble factors enhance neuronal density in the frontal cortex of depression-like mice

**DOI:** 10.1192/j.eurpsy.2024.580

**Published:** 2024-08-27

**Authors:** E. Markova, I. Rashchupkin, E. Shevela, E. Serenko, T. Amstislavskaya, A. Ostanin, E. Chernykh

**Affiliations:** ^1^State Research Institute of Fundamental and Clinical Immunology; ^2^State Research Institute of Neurosciences and Medicine, Novosibirsk, Russian Federation

## Abstract

**Introduction:**

Chronic inflammation in depression is associated with decreased levels of neurotrpohic factors and suppressed neurogenesis. We have previously shown that intranasal therapy with soluble factors from M2 macrophages polarized by interaction with apoptotic cells in serum deprivation conditions (M2(LS); LS - low serum) and characterized by anti-inflammatory and pro-regenerative activity leads to the correction of the behavioral pattern in mice with a depression-like state.

**Objectives:**

The present study focuses on the effect of M2(LS) macrophages on neuronal density in the frontal cortex and hippocampus of depression-like mice.

**Methods:**

Depressive-like state was formed in passive male mice (CBAxC57Bl/6)F1) as a result of repeated experience of defeat in agonistic interactions with aggressive partner during 20 days (the sensory contact model). Depression-like mice were then treated intranasally with M2(LS) macrophages conditioned medium for 7 days. After that, the number of mature neurons in the frontal cortex and hippocampus was assessed using Nissl staining.

**Results:**

The neuronal density in the pyramidal layer of the frontal cortex was significantly lower in depression-like mice than that in the intact control group of mice (p=0,047). At the same time, the number of neurons in the experimental group of mice that received soluble M2(LS) factors, was higher than that in depressive-like untreated control mice (p=0,003) and was comparable to that in the intact group of mice. At the same time  the neuronal density in the CA1 and CA3 hippocampal areas did not change in depression-like mice following intranasal treatment with conditioned medium of M2(LS) macrophages.

**Conclusions:**

The data obtained may indicate the neuroprotective effect of M2(LS) macrophages in the stress-induced depression model, which is realized through soluble factors and manifests itself in an increase of the pyramidal neurons density in the frontal cortex.

**Disclosure of Interest:**

None Declared

